# Effect of Different Fiber Sources as Additives to Wet Food for Beagle Dogs on Diet Acceptance, Digestibility, and Fecal Quality

**DOI:** 10.3390/vetsci10020091

**Published:** 2023-01-25

**Authors:** Amr Abd El-Wahab, Jan Berend Lingens, Julia Hankel, Christian Visscher, Cristina Ullrich

**Affiliations:** 1 Institute for Animal Nutrition, University of Veterinary Medicine Hannover, Foundation, Bischofsholer Damm 15, D-30173 Hannover, Germany; 2 Department of Nutrition and Nutritional Deficiency Diseases, Faculty of Veterinary Medicine, Mansoura University, Mansoura 35516, Egypt

**Keywords:** wet dog food, cellulose, lignocellulose, nutrient digestibility, fecal score

## Abstract

**Simple Summary:**

Obesity in dogs is a common problem that can have a negative impact on the health and welfare of these animals. For weight reduction, commercial diets with fibrous ingredients are produced. Because of the added fiber, energy intake can be reduced, and the feeling of satiety can be promoted. Cellulose is a common dietary fiber used mainly in powdered form; however, other processing forms or additives are available. This work aimed to investigate the influences of various types of fibers on palatability, apparent total tract digestibility, and fecal quality in dogs. Four different diets were fed to eight dogs for 14 days each. In addition to a basic diet without added fiber (control group), three experimental diets with the following fiber sources were fed: powdered cellulose, granulated cellulose, and lignocellulose. The study showed that all fiber supplements led equally to a reduction in energy intake compared with the basal diet, without affecting palatability. Fecal quality was not negatively affected by the fiber supplements; only wet fecal excretion was higher in the fiber groups than in the control groups. This study demonstrated that other fiber sources, such as granulated cellulose or lignocellulose, can be used as an alternative to cellulose without limitations.

**Abstract:**

In order to enhance the health and welfare of obese dogs and to facilitate the required loss of body weight, commercial diets are produced with fibrous ingredients. Cellulose is a common dietary fiber used mainly in powdered form. However, other processing forms and fibers are available as fibrous additives. This work aimed to test the effects of different fiber sources on apparent total tract digestibility and fecal quality in dogs. Four diets were fed to eight dogs (experimental design: 4 × 4 Latin square) for a 14-day period each. In addition to a basal diet (CO), three experimental diets varying in fiber sources were used: powdered cellulose (CE), granulated cellulose (GC), and lignocellulose (LC). Dogs fed the CO had lower crude fiber digestibility than those fed the other experimental diets (*p* < 0.0033). Dogs fed diets supplemented with fiber sources had lower gross energy digestibility (range: 76.2–77.3%) compared with those fed the CO (84.4%). In all groups, the fecal score (consistency and shape) ranged within the optimal values; solely wet fecal output was increased for the fiber groups compared with those on the CO. This study demonstrated that various sources of fiber such as GC and LC can be used as alternatives to CE without restrictions.

## 1. Introduction

In 2020, at least one pet was owned by about 88 million European households; approximately 24% of these households owned dogs [1]. Most companion animals now live in close relationship with their families, and are perceived as part of them [2]. Pet owners now demand improved nutritional food standards be offered to their animals as a result of this change [3]. The globally growing number of obese pets supports this development (in 2007, 52% and 55% of dogs and cats, respectively, compared with 56 % and 60 %, respectively in 2017 [4]). The causes of obesity are known to be multifactorial [5,6,7]. Moreover, this disease has long-term consequences for animal health, such as orthopedic disease, cardiorespiratory disorders, and gastrointestinal disorders [5,6,7]. Various factors may promote/increase pet obesity risk, such as inappropriate feeding, insufficient exercise, genetics, age, sex, and health conditions [8,9,10]. Thus, preventing the occurrence of obesity through weight management is key to maintaining the health and well-being of companion animals.

In most cases, increased exercise, combined with a controlled intake of carefully prepared food, results in successful weight loss [11]. Additionally, when it comes to weight loss plans for pets, high-fiber meals are frequently regarded as the ideal choice [12,13,14]. Because of their benefits to animal nutrition and health (reducing calorie consumption, enhancing satiety, and maintaining gastrointestinal health), pet food producers have manufactured canine foods using a number of fiber sources over the years [12,13,15,16,17,18]. The gastrointestinal (GI) microbiome represents an ecosystem of organisms that play important symbiotic roles in digestion, metabolism, nutrient absorption, and immunomodulation. Pet foods with specific fiber formulations that activate and nourish the canine GI microbiome and encourage selective microbial fermentation of the fiber in the gut may be particularly useful among dogs with chronic diarrhea [19,20].

So far, cellulose is mostly used as a source of dietary fiber for diets targeting weight loss, i.e., diets that are reduced in calories [21,22,23]. When trees are used to make paper pulp, the component cellulose is created. Cellulose is poorly fermented [24] and can reduce dry matter (DM) digestibility as well as organic matter digestibility in dogs [25]. It also increases fecal output according to several studies [26,27,28,29]. Furthermore, the higher the concentration of added cellulose, the more the digestion of organic matter is impaired, and the more the fecal output elevates [21,30]. Despite the benefits of cellulose for caloric dilution, product performance, and animal health, pet food manufacturers are looking to other types of fiber due to the product’s increased costs and limited consumer appeal [3]. 

Lignocellulose is a known sustainable energy source [31]. Additionally, as it is a component of plant cell walls, it is one of the most abundant and biologically renewable biomasses on earth [31]. Therefore, lignocellulose is used to manufacture biofuel [31]. From fresh naturally dried wood, lignocellulosic cellulose is mechanically extracted through fibrillation [32]. It can also be offered to dogs to replace traditional sources of fiber; nevertheless, to the best of our knowledge, very rarely have studies been performed in pets. The effects of lignocellulose on piglets have been studied [33]. The chemical construction of lignocellulose significantly diverges from the cellulosic structure, suggesting that its effect on the gastrointestinal tract is different from that of cellulose. Although lignocellulose consists of cellulose, which is the main structure, other components such as hemicellulose, pectin, and lignin are embedded therein [34]. Due to these features, lignocellulose is predicted to be lowly to moderately fermentable in canine intestines [34]. 

Another important aspect of including dietary fiber in pet food is the possible negative impact on palatability. For example, in a study by Koppel et al. [23], the intake of the feed with added fiber (in this case, sugar cane fiber and wheat bran were used) was lower. The dogs preferred the control diet without the additional fiber. The scientific literature provides very limited information on the impact of fiber on pet food acceptability. Therefore, additional research on this subject has to be conducted. The purpose of the presented experimental setup was to test the extent to which various fiber sources had an influence on apparent total tract digestibility, fecal quality, as well as acceptance in beagles, and whether differences were detectable between the different fibers.

## 2. Materials and Methods

### 2.1. Experimental Design and Diet Production

The University of Veterinary Medicine Hannover, Foundation, Hannover, Germany provided eight healthy, intact female Beagle dogs for this study. The average body weight (BW) of the beagles was about 11.1 kg and their age was within a range of 2–4 years. Once a week, the dogs were weighed and assessed for body condition scores (9-point scale [35]) prior to being fed. The animals were kept separately in kennels measuring 4.00 × 2.05 m, at constant room temperature, with light provided for 12 h daily. The trial was conducted using a 4 × 4 Latin square experimental design, in which the eight dogs were divided into two groups of four dogs each. Thereafter, the diets were changed within each group so that each dog received each of the four tested foods. The animals were accustomed to the food for nine days (d), and then feces were collected for five days so that each animal's apparent nutrient digestibility and fecal scores could be determined. Except for collection days, the dogs were provided time for interaction with conspecifics outside the kennels. Contact with humans was provided daily for the entire duration of the trial. Dogs had access to fresh water ad libitum. To measure the daily water intake (taking into account the evaporated fraction), each dog was provided with a weighed container of sufficient size filled with fresh water. At the end of each day, the remaining amount of water was weighed and subtracted from the amount weighed in the morning. The amount of food received by the dogs was calculated using the equation for the daily energy requirements of adult dogs (0.45 MJ metabolizable energy × BW^0.75/^d) [36], and the diets consisted of the same nutrient content, except for the source of dietary fiber. The animals were fed once per day. The daily food intake of all dogs averaged at 649 ± 33 g of wet food. The amount of food offered and that remaining in the bowl was noted daily after each meal to determine the amount of food consumed. The base meal was a wet commercial diet (Royal Canin^®^ Veterinary Diet Senior Consult Mature; ROYAL CANIN Tiernahrung GmbH & Co. KG, Cologne, Germany) supplied to the control group (CO). The other three experimental diets were made by adding three different fiber sources to the basic diet. The experimental fiber sources were powdered cellulose (JELUCEL, CE), granulated cellulose (GC), and lignocellulose (JELUVET, LC). The fiber sources were commercially purchased (JELU-WERK J. Ehrler GmbH & Co. KG, Rosenberg, Germany). The amount of each was also determined on the basis of BW and consisted of 2 g/kg. The average amount of fiber additive was about 22.2 ± 1.50 g. 

### 2.2. Chemical Analysis

The chemical analysis of all samples was carried out in duplicate. Samples from each collection period were defrosted and mixed to obtain one pooled sample per week and experimental diet. In order to analyze the dried feed material, it was ground through a 1 mm sieve (Retsch ZM200, Haan, Germany). The Association of German Agricultural Analytical and Research Institutes e.V. (VDLUFA) [37] procedures were used to determine the nutrients in the diets and fecal samples (Table 1). To measure the calcium concentration, an atomic absorption spectrometer (Solaar M-Serie Atomic Absorption Spectometer, Cambridge, England) was used in accordance with the Association of Official Analytical Chemists (AOAC) [38], while the phosphorus level was photometrically measured (Spectrophotometer UV-1900 i, Schimadzu Corporation, Kyoto, Japan) using the vanadate molybdate method, as described by Gericke and Kurmies [39]. The metabolizable energy content of the diets was calculated, as recommended by the National Research Council [36], according to the following equation: (MJ/kg) = 0.01674 × crude protein + 0.03767 × crude fat + 0.1674 × nitrogen-free extract. 

The procedure for the determination of total dietary fiber (TDF) was based on the methods of Lee et al. [40] and Prosky et al. [41]. Briefly, 1 g of the dried food sample was subjected to sequential enzymatic digestion by heat-stable α-amylase, protease, and amyloglucosidase. After filtering out the insoluble components, the soluble fiber was precipitated with ethanol after adding distilled water to the filtrate. To determine the TDF, the residue was filtered, dried, and weighed. The TDF value was corrected for protein and ash content.

### 2.3. Scores for Food Intake and Apparent Total Tract Digestibility 

The food intake scoring (palatability and speed of food intake) was classified into three groups in accordance with Zahn [42]. Briefly, score 1 indicates the lowest level of acceptance, score 2 indicates a moderate level of acceptance, and score 3 indicates the highest level of acceptance. After the adaption phase (9 d), the collection phase began (for 5 d), during which the feces excreted daily were collected. Following the weighing of the fresh feces, a subsample of 10% of the feces per animal per day was analyzed for DM content. Subsequently, the remaining fecal samples were stored at −20 °C. At the end of the study, the five-day fecal samples from each dog were thawed and homogenized for further analyses. The apparent total tract digestibility (%) was computed by multiplying ((food − feces)/food) by 100.

### 2.4. Fecal Scores

**Figure 1:**
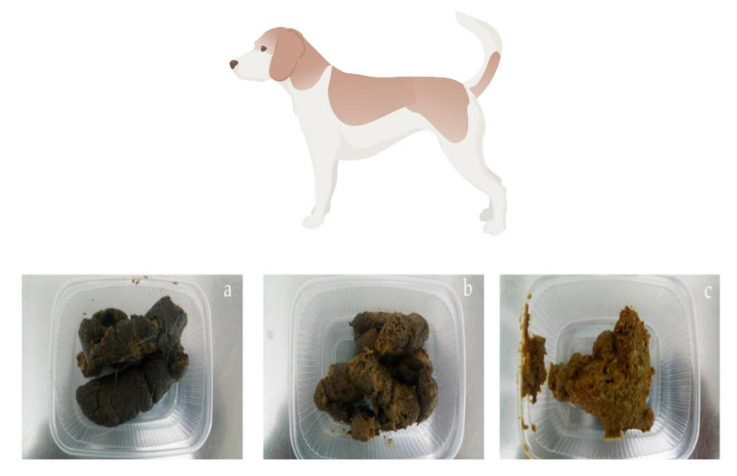
Fecal scores in accordance with Moxham [43] study: a = score 2 (solid, well formed); b = score 3 (soft, still formed); c = score 4 (pasty, slushy); Photo: Abd El-Wahab, A. ^©^TiHo (figure was created with BioRender.com on 22 May 2022).

Fecal consistency scores were calculated using a five-point scale: 1 for extremely hard feces; 2 for solid, well-formed, 'optimum' feces; 3 for soft, still-forming feces; 4 for pasty, slushy feces; and 5 for watery diarrhea, in accordance with Moxham [43], as shown in Figure 1. The fecal form scoring system devised by Abd El-Wahab et al. [44] involves a four-point scale (where 1 represents an individual fecal mass; 2, strong constrictions at the ‘optimal’ fecal surface; 3, cracks at the fecal surface; and 4, shapelessness), which was used to describe the shape of feces.

### 2.5. Statistical Analysis

The statistical evaluation was carried out with Statistical Analysis System (SAS) Enterprise Guide 7.1 (SAS Institute Inc., Cary, NC, USA). Mean values, as well as the standard deviation (SD) of the mean, were computed for all parameters. A Shapiro–Wilk test for normal distribution was performed for continuous data, and normally distributed data were checked for significant differences with the parametric Ryan–Eino–Gabriel–Welsch test (one-way ANOVA). For non-normally distributed data, the nonparametric Kruskal–Wallis test was performed, followed by the non-parametric pairwise Wilcoxon two-sample test. The significance level was set at *p* < 0.05.

## 3. Results

During the entire time of the trial, the dogs showed good overall health status. Throughout the study, the BW of the dogs was comparable among the experimental groups (*p* > 0.05). During feed intake, high acceptance was observed, thus achieving a high score (range: 1.00–1.43; *p* = 0.3178), and no refusals were observed. Additionally, the water intake was comparable among the groups (range: 135–223 g; *p* = 0.6962). Moreover, the BW of the dogs remained unchanged throughout the trial (average 11.4 kg BW). Additionally, the BCS values were maintained (average body condition score: 4.88).

### 3.1. Apparent Nutrient Digestibility

The results of the apparent nutrient digestibility are shown in Table 2. No significant differences were observed for the apparent digestibility of crude ash, crude fat, and crude protein among the groups. The dogs fed the basic diet (CO) without supplementation showed significantly lower crude fiber digestibility (22.6 %) compared with the other groups. No significant differences were observed in the apparent digestibility of calcium, phosphorus, or magnesium among the three groups (Table 2). Additionally, the supplementation of various fiber sources resulted in significantly lower digestibility of gross energy (range: 76.2–77.3%) among the dogs in comparison with those animals fed the nonsupplemented diet (84.4%).

### 3.2. Fecal Quality

The fecal consistency scores (Table 3) did not differ among the groups (range: 2.02–2.25; *p* = 0.2670). Additionally, supplementing the basic diet with various fiber sources did not result in differences in the fecal form scores among the groups (range: 2.01–2.16; *p* = 0.5412). The quantity of daily wet feces was significantly affected by the addition of different fiber sources (range: 137–143 g/d) compared with that of dogs fed the nonsupplemented basic diet (99.1 g/d). No significant differences were noted in the fecal DM content among the test groups (range: 31.4–35.6 %; *p* = 0.0786).

## 4. Discussion

In the present trial, the fiber digestibility was significantly affected by the fiber quantity, irrespective of its source. Several studies have demonstrated that the amount and quality of different fiber sources can influence the digestibility of nutrients [16,25,45,46]. This fact was demonstrated in the present work by the increased excretion of undigested and unfermented matter. 

Similar protein digestion in dogs was not affected by a change in the supplemented fiber (beet pulp or corn fiber,) or the amount of fiber used studies were performed in other dog breeds and other countries [47]. In a study conducted by Guevara et al. [48], (TDF 8.40% to 10.2%). In the present study and in a study by Kienzle et al. [49], it was found that increasing dietary fiber content, more undigested organic material was excreted by the animals. Nevertheless, not all studies could confirm such an effect of dietary fiber sources [50]. In contrast to our results, in a study by Beloshapka et al. [51], the digestibility of crude protein decreased as total dietary fiber consumption increased in dogs. In their study, resistible corn starch, which is fermentable by gut microbiota, was used in increasing concentrations (0% to 4%). However, the authors did not attribute the reduced protein digestibility to the increased fiber content, but suggested that it could be related to an increase in microbial activity and fecal nitrogen excretion.

In the current work, 2% of each of the various dietary fibers was added to commercial food. The idea behind the use of equal amounts of fiber was achieving a uniformly elevated dietary fiber level and ensuring as equal a composition as possible of the remaining ingredients of the basic feed. Therefore, our hypothesis was that the type of dietary fiber was the only factor contributing to the findings, not differences resulting from shifts in the proportion of other dietary components. The commercial diets used for weight maintenance and weight loss most commonly contain high levels of dietary fiber. Consequently, it was necessary to simulate these diets through the addition of 2% of the respective fibers that were to be tested. This percentage has also been used in previous studies demonstrating that nutrient digestibility is thereby not reduced to an undesirable extent and that positive effects on fecal quality, among others, can already be observed [49,52]. In the present study, no differences in food intake occurred relative to the added fibers (an average of 649 g of the experimental diet was consumed). Refusal of feed intake did not occur over the course of the entire trial. 

A key factor to consider when assessing dog foods is fecal quality. This study found that neither the addition of fiber alone nor the kind of fiber element to the diet had any effect on the fecal consistency score (average: 2.16). Similarly, there was no difference in the fecal shape scores between dogs that received the control food and those in the fiber-added groups. Additionally, there were no differences among the groups given various fiber sources (average: 2.10). Dogs supplied with fiber-rich diets had significantly higher wet fecal output (average: 140 g/d), regardless of the fiber source, than those offered a nonsupplemented diet (99.1 g/d). Nevertheless, the fecal DM did not follow the same trend as wet fecal output and remained without differences at a range of 31.4–35.6%. 

The DM content in the feces of pets can be significantly influenced by numerous different factors. One factor, for example, is the fermentation activity of fibers. The fermentation activity and the moisture content of dog feces have been shown to have strong positive associations [16,26,53]. This finding might be connected to fiber's ability to add bulk, and it seems to be most strongly connected to sources of insoluble fiber that are both poorly fermentable and have a high capacity to bind water [26]. 

Short-chain fatty acids are often produced in greater quantities by the gastrointestinal microbial fermentation of soluble fiber (mainly acetate, propionate, and butyrate). Pigs’ digestive tracts absorb more water due to short-chain fatty acids. However, the overdosing of butyrate might induce an osmotic effect, resulting in increased fecal moisture content in pigs [54]. Guevara et al. [48] offered food to dogs containing beet pulp and different sources of corn fiber. They found a decrease in fecal DM when beet pulp (moderately fermentable fiber) was added to the diet compared with when the tested corn fiber was added. While the TDF content of these fiber sources was similar, the soluble fiber content of the corn fiber was much lower than that of the beet pulp [48], thereby confirming the idea that increasing the amount of soluble fiber in the diet, and thus in the intestinal lumen, may alter the nature of water flow and decrease the DM content of feces. 

Moreover, the water intake of animals may be reduced through the addition of dietary fibers, due to their different water-binding capacities. Thus, in addition to the reduced water intake, as well as the aforementioned alteration in water flow, the dry matter of the feces may be reduced. In their study, Silvio et al. [46] used experimental diets for dogs with varying proportions of cellulose (as an insoluble fiber) and pectin (as a soluble fiber). Then, digestibility was measured at the ileum and total tract. They reported a decrease in fecal DM percentage, as the pectin content of the diet increased at the expense of that of the cellulose. This supports the hypothesis that the fermentation of soluble fibers could increase fecal water content. 

Because the fiber used in the present study was insoluble [46], the DM content in the feces was not affected. This is explained by the low water-binding capacity of cellulose [54]. Lignocellulose is also considered an insoluble fiber, and, like cellulose, has a low water-binding capacity [33]. Therefore, the DM content in the feces was not negatively influenced either. Moreover, in the present study, the amount of wet fecal mass per day was significantly affected by the content of the dietary fiber. In agreement with our data, dietary fiber is known to affect the volume of fecal excretion negatively [26,28,29].

## 5. Conclusions

Supplementing these three fiber sources did not affect the fecal consistency and/or form scores. Independent of the fiber source, increasing the amount of dietary fiber resulted in a significant increase in the wet fecal output. In view of the results of this study, lignocellulose can be used as an alternative to cellulose as a fiber source in wet dog food. As lignocellulose reduces gross energy digestibility to the same extent as cellulose, it can also be used in dietary feed for overweight dogs. When feeding the regular amount of feed, less energy is utilized when using these fibers.

## Figures and Tables

**Table 1:** Chemical composition of the basic diet fed to Beagle dogs (g/kg DM).

Parameter	Unit	Content
DM	g/kg fed	232
Crude ash	g/kg DM	58.9
Crude protein		393
Crude fat		207
Crude fiber		41.2
TDF		113
Nitrogen-free extract		301
Calcium		7.80
Phosphorus		8.40
Sodium		3.40
Potassium		9.30
Magnesium		0.80
Zinc	mg/kg DM	153
Selenium		1.00
Metabolizable energy	MJ/kg DM	18.2

**Table 2:** Apparent digestibility of selected parameters (%) of diets offered to dogs with various fiber sources (mean ± SD).

Digestibility	CO *	CE *	GC *	LC *	*p*-Value
Crude ash	48.1 ± 6.20	46.1 ± 5.80	44.3 ± 5.10	45.5 ± 5.80	0.6087
Crude fiber	−22.6 ^b^ ± 20.3	−6.25 ^a^ ± 8.25	−4.44 ^a^ ± 9.90	3.06 ^a^ ± 8.42	0.0033
Crude fat	97.5 ± 0.10	97.7 ± 0.30	97.7 ± 0.30	97.8 ± 0.20	0.1134
Crude protein	81.6 ± 2.10	81.1 ±3.10	80.9 ± 2.40	80.4 ± 2.10	0.8119
Calcium	10.0 ± 13.6	10.5 ± 7.10	7.37 ± 9.29	7.35 ± 10.8	0.8886
Phosphorus	51.1 ± 6.80	51.8 ± 3.10	49.1 ± 4.70	50.4 ± 6.70	0.7934
Magnesium	24.8 ± 11.6	23.5 ± 5.49	20.2 ± 8.24	22.7 ± 9.18	0.7728
Gross energy	84.4 ^a^ ± 1.70	76.7b ± 2.60	77.3 ^b^ ± 2.30	76.2 ^b^ ± 2.30	<0.0001

^a, b^ Means in a row with different superscripts differ significantly (*p* < 0.05); * CO: basic diet/control group, CE: cellulose group, GC: granulated cellulose group, and LC: lignocellulose group.

**Table 3:** Fecal characteristics of dogs fed diets with different fiber sources (mean ± SD).

Parameters	Unit	CO *	CE *	GC *	LC *	*p*-Value
Fecal consistency score	1–5	2.19 ± 0.21	2.25 ± 0.35	2.16 ± 0.18	2.02 ± 0.12	0.267
Fecal form score	1–4	2.12 ± 0.20	2.16 ± 0.30	2.11 ± 0.23	2.01 ± 0.06	0.5412
Fecal amount	g wet/d	99.1 ^b^ ± 11.0	143 ^a^ ± 29.0	137 ^a^ ± 22.6	141 ^a^ ± 16.1	0.0005
DM content	%	31.4 ± 2.19	35.2 ± 4.24	35.6 ± 2.05	34.4 ± 3.21	0.0786

^a, b^ Means in a row with different superscripts differ significantly (*p* < 0.05); * CO: basic diet/control group, CE: cellulose group, GC: granulated cellulose group, and LC: lignocellulose group.

## Data Availability

The data presented in this study are available in this manuscript.
